# Medical conditions at enrollment do not impact efficacy and safety of the adjuvanted recombinant zoster vaccine: a pooled post-hoc analysis of two parallel randomized trials

**DOI:** 10.1080/21645515.2019.1627818

**Published:** 2019-06-28

**Authors:** Lidia Oostvogels, Thomas C. Heineman, Robert W. Johnson, Myron J. Levin, Janet E. McElhaney, Peter Van den Steen, Toufik Zahaf, Alemnew F. Dagnew, Roman Chlibek, Javier Diez-Domingo, Iris S. Gorfinkel, Caroline Hervé, Shinn-Jang Hwang, Hideyuki Ikematsu, George Kalema, Himal Lal, Shelly A. McNeil, Tomas Mrkvan, Karlis Pauksens, Jan Smetana, Daisuke Watanabe, Lily Yin Weckx, Anthony L. Cunningham

**Affiliations:** a GSK, Wavre, Belgium; b GSK, King of Prussia, PA, USA; c Faculty of Health Sciences, University of Bristol, Bristol, UK; d Departments of Pediatrics and Medicine, University of Colorado, Anschutz Medical Campus, Aurora, CO, USA; e Health Sciences North Research Institute, Sudbury, Ontario, Canada; f GSK, Rockville, MD, USA; g Faculty of Military Health Sciences, University of Defense, Hradec Kralove, Czech Republic; h Fundación para el Fomento de la Investigación Sanitaria y Biomédica, Valencia, Spain; i PrimeHealth Clinical Research, Toronto, Ontario, Canada; j Department of Family Medicine, Taipei Veterans General Hospital and National Yang Ming University School of Medicine, Taipei, Taiwan; k Japan Physicians Association, Tokyo, Japan; l Keyrus Biopharma, Waterloo, Belgium, on behalf of GSK; m Canadian Center for Vaccinology, IWK Health Center and Nova Scotia Health Authority, Dalhousie, University, Halifax, Canada; n Department of Infectious Diseases, Uppsala University Hospital, Uppsala, Sweden; o Department of Dermatology, Aichi Medical University, Nagakute, Japan; p Department of Pediatrics, Federal University of Sao Paulo, Sao Paulo, Brazil; q The Westmead Institute for Medical Research, Westmead, University of Sydney, Sydney, NSW, Australia

**Keywords:** Varicella-zoster virus, adjuvanted recombinant zoster vaccine, vaccine efficacy, vaccine safety, underlying chronic disease, comorbidity

## Abstract

In two pivotal efficacy studies (ZOE-50; ZOE-70), the adjuvanted recombinant zoster vaccine (RZV) demonstrated >90% efficacy against herpes zoster (HZ).

Adults aged ≥50 or ≥70 years (ZOE-50 [NCT01165177]; ZOE-70 [NCT01165229]) were randomized to receive 2 doses of RZV or placebo 2 months apart. Vaccine efficacy and safety were evaluated post-hoc in the pooled (ZOE-50/70) population according to the number and type of selected medical conditions present at enrollment.

At enrollment, 82.3% of RZV and 82.7% of placebo recipients reported ≥1 of the 15 selected medical conditions. Efficacy against HZ ranged from 84.5% (95% Confidence Interval [CI]: 46.4–97.1) in participants with respiratory disorders to 97.0% (95%CI: 82.3–99.9) in those with coronary heart disease. Moreover, efficacy remained >90% irrespective of the number of selected medical conditions reported by a participant.

As indicated by the similarity of the point estimates, this post-hoc analysis suggests that RZV efficacy remains high in all selected medical conditions, as well as with increasing number of medical conditions. No safety concern was identified by the type or number of medical conditions present at enrollment.

## Introduction

Herpes zoster (HZ) results from reactivation of latent varicella-zoster virus (VZV) in sensory ganglia long after primary infection. Worldwide, the incidence of HZ ranges between 3–5 cases per 1,000 person-years in the general population and increases markedly with age, with more than two-thirds of HZ cases occurring in adults over 50 years of age (YOA).^1,2^

Medical conditions previously identified as increasing the risk of HZ include systemic lupus erythematosus, rheumatoid arthritis, inflammatory bowel disease, chronic obstructive pulmonary disease, asthma, chronic kidney disease, renal failure, hypertension, diabetes mellitus (predominantly type I), depression, and spinal disc herniation.^3–5^ The increase in HZ risk associated with some of these conditions may result from the immunosuppressive therapy prescribed to treat the disease and/or from underlying cell-mediated immunosuppression associated with the disease.^4^

Vaccination decreases the risk of HZ.^6–9^ The adjuvanted recombinant zoster vaccine (RZV, *Shingrix*), containing a truncated form of VZV glycoprotein E and the AS01_B_ adjuvant system, demonstrated 97.2% and 91.3% vaccine efficacy (VE) against HZ in adults ≥50 (ZOE-50) and ≥70 YOA (ZOE-70), respectively, over an approximate 4-year follow-up period. Efficacy remained >90% among participants ≥80 YOA.^7,8^ RZV also demonstrated an acceptable safety profile.^7,8,10^ It is currently licensed in multiple countries for the prevention of HZ in adults ≥50 YOA.

Although adults with a life expectancy of less than 4 years, with immunosuppressive conditions (e.g., resulting from malignancy or HIV infection) or requiring treatment with immune-modifying drugs (e.g., medications used during cancer chemotherapy or organ transplantation) were excluded from entry into the ZOE-50/70 trials,^7,8^ the eligibility criteria allowed enrollment of adults with medical conditions that are common in the general older adult population. The study population was therefore considered broadly representative of the general older adult population. The two studies were conducted in parallel at the same sites and in an identical manner, allowing the analysis of pooled data from both trials. Adults ≥70 YOA were randomly assigned to the ZOE-50 or ZOE-70 study before being randomized to a study group.

The objective of this post-hoc pooled analysis is to evaluate RZV efficacy against the first or only episode of HZ and to examine RZV safety in participants with selected medical conditions at enrollment.

## Methods

The ZOE-50/70 studies were phase III, randomized, observer-blind, controlled trials conducted in parallel in 18 countries in Europe, North America, Latin America, Asia and Australia in adults ≥50 YOA (NCT01165177) and ≥70 YOA (NCT01165229). Participants were randomized 1:1 to receive 2 doses of either RZV or saline placebo 2 months (M) apart.^7,8^ Protocol summaries are available for both studies at http://www.gsk-clinicalstudyregister.com (studies 110390 and 113077). Anonymized individual participant data and study documents can be requested for further research from www.clinicalstudydatarequest.com.

Persons with a confirmed or suspected immunosuppressive or immunodeficient conditions resulting from disease (e.g., malignancy, human immunodeficiency virus infection) or immunosuppressive/cytotoxic therapy (e.g., medications used during cancer therapy, organ transplantation or to treat autoimmune disorders) were excluded. Persons who had received immunosuppressants or immune-modifying drugs for >15 consecutive days within 6 months prior to the first vaccine dose, were also excluded (prednisone <20 mg/day, or equivalent, and inhaled/topical steroids were allowed). Full inclusion and exclusion criteria have previously been published.^7,8^

Medical conditions of the approximately 30,000 participants were recorded at enrollment in the ZOE-50/70 trials, and those most frequently reported in the ZOE-70 trial were selected and applied for analysis utilizing data from both trials, since the prevalence of most underlying medical conditions increases with age. Efficacy and safety analyses were performed post-hoc in the pooled ZOE-50/70 population (i) according to medical conditions reported by at least 500 participants from either arm of the ZOE-70 study at enrollment (considered to provide an adequate sample size for the purpose of a descriptive analysis), and according to additional medical conditions reported by less than 500 participants in the ZOE-70 study at enrollment but associated with an increased risk of HZ (asthma, depression, respiratory disorders, and renal disorders),^3–5^ and (ii) according to the number of selected medical conditions reported by study participants at enrollment. For the purpose of the analysis, medical conditions were defined as individual Medical Dictionary for Regulatory Authorities preferred terms (PTs) or grouped PTs representing conditions of similar pathophysiological origin. Details on PT grouping are provided in the Supplementary Table 1. Participants reporting more than one of the PTs within the 15 selected medical conditions were only counted once for the respective analyses.

For the analysis of VE, a suspected HZ case was defined as new unilateral rash with pain that had no alternative diagnosis. Participants were followed for at least 90 days after the onset of HZ or until the rash resolved and the participant was pain-free for 4 weeks. Suspected HZ cases were evaluated and confirmed as described previously by polymerase-chain-reaction or an adjudication committee.^7,8^ Efficacy was assessed similar to the primary analysis on the pooled modified total vaccinated cohort (mTVC).^7,8^ This included all participants from the pooled TVC who had received both doses of vaccine/placebo and who had not developed a confirmed HZ case prior to 30 days post-dose 2. VE was defined as 1 minus the ratio of HZ incidence in the RZV group to that in the placebo group.

For assessment of safety, serious adverse events (SAEs) were recorded for all participants for up to 12 months post-dose 2; any events resulting in death, and potentially immune-mediated diseases (pIMDs) were evaluated in all participants over the entire study period. Safety was evaluated in the pooled TVC from the ZOE-50/70 studies, which included all participants administered with at least one dose of RZV.

## Results

**Figure 1.**
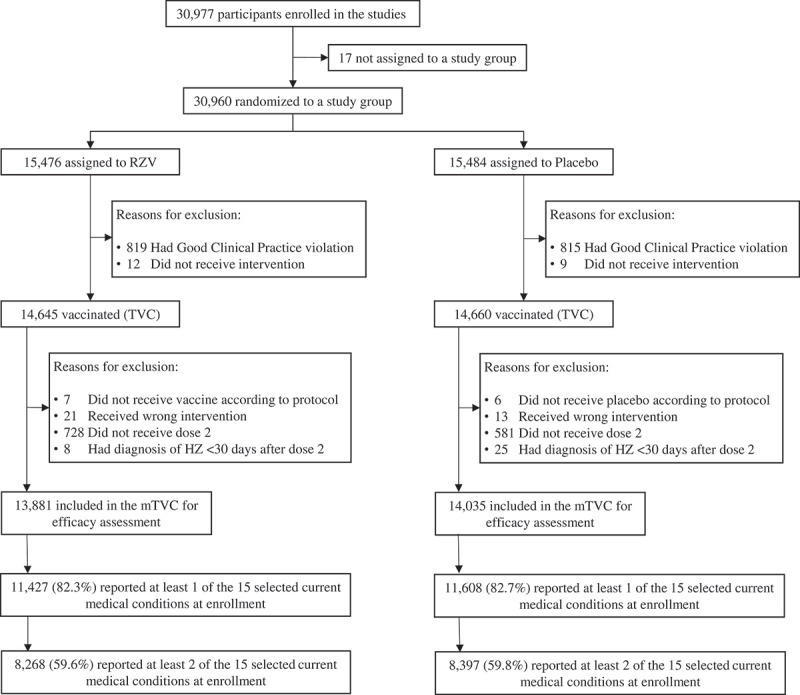
Participant flow (pooled ZOE-50/70 studies) RZV = participants receiving the adjuvanted recombinant zoster vaccine; Placebo = participants receiving placebo; ZOE-50/70 = RZV efficacy studies in adults ≥50 YOA (NCT01165177) and ≥70 YOA (NCT01165229), respectively; (m)TVC = (modified) total vaccinated cohort; HZ, herpes zoster.

Of the 30,977 participants enrolled in the ZOE-50/70 studies, 13,881 RZV and 14,035 placebo recipients were included in the mTVC. Of these, 82.3% of RZV recipients and 82.7% of placebo recipients had at least 1 of the 15 selected medical conditions at enrollment. A majority (RZV: 59.6%, Placebo: 59.8%) had 2 or more (Figure 1). In the pooled mTVC, demographic characteristics, including proportions of participants reporting each of the selected medical conditions, were balanced between study groups (Table 1).

**Table 1.** Demographic characteristics (pooled ZOE-50/70 modified Total Vaccinated Cohort).

	RZV (N = 13,881)	Placebo (N = 14,035)
**Age (years)**		
Mean age at first dose ± SD	68.5 ± 9.8	68.5 ± 9.8
**Gender, n (%)**		
Female	8,044 (57.9)	8,178 (58.3)
Male	5,837 (42.1)	5,857 (41.7)
**Geographic ancestry, n (%)**		
White – Caucasian/European	10,321 (74.4)	10,403 (74.1)
Asian – East Asian	1,908 (13.7)	1,926 (13.7)
Asian – Japanese Heritage	575 (4.1)	591 (4.2)
African/African American	200 (1.4)	183 (1.3)
Other	877 (6.3)	932 (6.6)
**Selected medical conditions present at enrollment, n (%)**		
Hypertension	7,206 (51.9)	7,226 (51.5)
Osteoarthritis and/or vertebral disorders	4,951 (35.7)	5,032 (35.9)
Dyslipidemia	4,628 (33.3)	4,707 (33.5)
Diabetes	2,350 (16.9)	2,372 (16.9)
Osteoporosis/Osteopenia	1,481 (10.7)	1,528 (10.9)
Gastroesophageal reflux disease	1,334 (9.6)	1,313 (9.4)
Sleep disorder	1,304 (9.4)	1,309 (9.3)
Prostatic diseases	1,244 (9.0)	1,285 (9.2)
Hypothyroidism	1,167 (8.4)	1,147 (8.2)
Depression	1,017 (7.3)	987 (7.0)
Coronary heart disease	1,003 (7.2)	1,055 (7.5)
Cataract	782 (5.6)	800 (5.7)
Asthma	646 (4.7)	689 (4.9)
Respiratory disorders^#^	614 (4.4)	560 (4.0)
Renal disorders	308 (2.2)	300 (2.1)

RZV = participants receiving the adjuvanted recombinant zoster vaccine; Placebo = participants receiving placebo; ZOE-50/70 = RZV efficacy studies in adults ≥50 YOA (NCT01165177) and ≥70 YOA (NCT01165229); N = number of participants in the pooled modified total vaccinated cohorts; n (%) = number (percentage) of participants in each category; SD = standard deviation.

^#^Other than asthma.

Medical conditions in order of decreasing frequency included hypertension, osteoarthritis and vertebral disorders, and dyslipidemia. The most frequently reported conditions known to increase HZ risk included in this analysis were depression and asthma (Table 1).

Efficacy against HZ ranged from 84.5% (95% Confidence Interval [CI]: 46.4–97.1) in participants with respiratory disorders to 97.0% (95% CI: 82.3–99.9) in those with coronary heart disease at enrollment. Efficacy was 88.8% (95% CI: 63.6–97.8) in participants with asthma, and 91.2% (95% CI: 81.1–96.6) in those with diabetes. The only medical condition that RZV efficacy did not achieve statistical significance was renal disorders (VE: 86.6% [95% CI: −4.5–99.7]). The low number of participants with this condition limited the statistical power to assess VE. Overall, RZV efficacy was >90% irrespective of the number of the selected medical conditions reported at enrollment by a participant (Table 2).

**Table 2.** Vaccine efficacy against first or only episode of herpes zoster in ZOE-50/70 participants reporting at least 1 of the 15 selected medical conditions at enrollment (pooled modified Total Vaccinated Cohort).

	RZV				Placebo				
	N	n	Cumulative follow-uppy	Rate of HZ cases/ 1,000 py	N	n	Cumulative follow-up py	Rate of HZ cases/ 1,000 py	Vaccine efficacy % (95% CI)
**Selected medical conditions present at enrollment**									
Hypertension	7,206	21	27,202.9	0.8	7,226	254	26,752.4	9.5	91.9 (87.3–95.1)
Osteoarthritis and/or vertebral disorders	4,951	16	18,732.8	0.9	5,032	178	18,604.4	9.6	91.1 (85.1–95.0)
Dyslipidemia	4,628	15	17578.0	0.9	4,707	169	17,507.2	9.7	91.2 (85.1–95.2)
Diabetes	2,350	7	8,723.8	0.8	2,372	80	8,652.7	9.2	91.2 (81.1–96.6)
Osteoporosis/Osteopenia	1,481	5	5,551.7	0.9	1,528	72	5,552.1	13.0	92.9 (82.7–97.8)
Gastroesophageal reflux disease	1,334	6	5,009.7	1.2	1,313	44	4,816.2	9.1	86.9 (69.0–95.4)
Sleep disorder	1,304	4	4,899.3	0.8	1,309	56	4,803.3	11.7	93.1 (81.4–98.2)
Prostatic diseases	1,244	2	4,648.4	0.4	1,285	50	4,667.0	10.7	96.1 (85.1–99.5)
Hypothyroidism	1,167	4	4,387.0	0.9	1,147	28	4,241.0	6.6	86.2 (60.4–96.5)
Depression	1,017	2	3,767.1	0.5	987	29	3,567.5	8.1	93.4 (74.1–99.2)
Coronary heart disease	1,003	1	3,773.8	0.3	1,055	35	3,912.8	8.9	97.0 (82.3–99.9)
Cataract	782	4	2,964.7	1.3	800	41	2,931.0	14.0	90.4 (73.4–97.5)
Asthma	646	3	2,420.9	1.2	689	28	2,575.8	10.9	88.8 (63.6–97.8)
Respiratory disorders^#^	614	3	2,220.5	1.4	560	17	1,944.4	8.7	84.5 (46.4–97.1)
Renal disorders	308	1	1,064.8	0.9	300	7	1,001.5	7.0	86.6 (-4.5–99.7)
**Number of selected medical conditions present at enrollment**									
1	3,159	5	12,269.2	0.4	3,211	109	12,213.4	8.9	95.4 (89.0–98.5)
2	3,080	7	11,797.1	0.6	3,117	97	11,746.4	8.3	92.8 (84.7–97.2)
3	2,316	8	8,803.7	0.9	2,455	88	9,162.6	9.6	90.5 (80.5–96.0)
At least 3	5,188	19	19,417.0	1.0	5,280	199	19,338.4	10.3	90.5 (84.8–94.4)
At least 4	2,872	11	10,613.3	1.0	2,825	111	10,175.8	10.9	90.6 (82.4–95.4)
At least 5	1,406	5	5,132.5	1.0	1,350	52	4,742.4	11.0	91.2 (78.0–97.3)
At least 6	569	2	2,039.2	1.0	551	20	1,910.1	10.5	90.9 (62.5–99.0)

RZV = participants receiving the adjuvanted recombinant zoster vaccine; Placebo = participants receiving placebo; ZOE-50/70 = RZV efficacy studies in adults ≥50 YOA (NCT01165177) and ≥70 YOA (NCT01165229), respectively; N = number of participants in each category; n = number of confirmed first or only herpes zoster case; HZ, herpes zoster; CI = confidence interval; py = person years. The follow-up period was censored at the first occurrence of a confirmed HZ episode.

^#^Other than asthma.

In RZV or placebo recipients reporting at least 1 of the 15 selected medical conditions at enrollment, the proportion of participants experiencing SAEs or deaths was highest among those with chronic conditions such as renal disorders, respiratory disorders, or coronary heart disease. The numbers of SAEs, deaths, or pIMDs were similar in the vaccine and placebo groups for each of the medical conditions (Table 3). The frequency of SAEs and deaths increased with the number of medical conditions present at enrollment; however, the frequency of pIMDs did not. Frequencies of SAEs, deaths, and pIMDs were balanced between RZV and placebo recipients reporting any number of these conditions (Table 3).

**Table 3.** Safety of RZV in ZOE-50/70 participants reporting at least 1 of the 15 selected medical conditions at enrollment (pooled Total Vaccinated Cohort).

			Reported from dose 1 until 1 year post-dose 2		Reported during the whole post-vaccination period			
	Number of participants in the TVC		SAEs		Deaths		pIMDs	
	RZV	Placebo	RZV % (95% CI)	Placebo % (95% CI)	RZV % (95% CI)	Placebo % (95% CI)	RZV % (95% CI)	Placebo % (95% CI)
**Selected medical conditions present at enrollment**								
Hypertension	7,609	7,556	12.5 (11.8–13.3)	12.6 (11.9–13.4)	5.7 (5.2–6.3)	6.3 (5.7–6.8)	1.2 (1.0–1.5)	1.2 (0.9–1.4)
Osteoarthritis and/or vertebral disorders	5,212	5,258	12.5 (11.6–13.4)	13.2 (12.3–14.1)	5.1 (4.5–5.7)	5.5 (4.9–6.1)	1.5 (1.2–1.8)	1.7 (1.4–2.1)
Dyslipidemia	4,904	4,953	12.1 (11.2–13.0)	12.1 (11.2–13.0)	4.7 (4.1–5.3)	4.5 (4.0–5.1)	1.1 (0.8–1.4)	1.6 (1.3–2.0)
Diabetes	2,480	2,502	15.2 (13.8–16.7)	15.4 (14.0–16.9)	7.3 (6.3–8.4)	8.3 (7.2–9.4)	1.0 (0.6–1.4)	1.3 (0.9–1.8)
Osteoporosis/Osteopenia	1,568	1,592	11.9 (10.4–13.6)	12.9 (11.3–14.6)	5.2 (4.1–6.4)	5.2 (4.2–6.4)	1.3 (0.8–2.0)	1.8 (1.2–2.5)
Gastroesophageal reflux disease	1,407	1,374	14.4 (12.6–16.4)	15.1 (13.2–17.1)	4.3 (3.3–5.5)	5.6 (4.4–7.0)	1.1 (0.7–1.8)	2.3 (1.6–3.3)
Sleep disorder	1,379	1,389	13.6 (11.9–15.6)	13.0 (11.2–14.8)	6.6 (5.3–8.0)	7.3 (6.0–8.8)	1.5 (0.9–2.3)	1.7 (1.1–2.5)
Prostatic diseases	1,319	1,353	16.6 (14.6–18.7)	16.5 (14.5–18.6)	8.3 (6.9–10.0)	7.8 (6.4–9.3)	0.8 (0.4–1.5)	1.5 (0.9–2.3)
Hypothyroidism	1,218	1,208	11.2 (9.5–13.2)	12.3 (10.5–14.3)	3.4 (2.5–4.6)	4.0 (2.9–5.2)	1.9 (1.2–2.8)	1.8 (1.1–2.7)
Depression	1,095	1,070	14.2 (12.2–16.5)	15.4 (13.3–17.7)	6.1 (4.8–7.7)	5.3 (4.1–6.8)	1.0 (0.5–1.8)	1.1 (0.6–2.0)
Coronary heart disease	1,065	1,097	20.4 (18.0–22.9)	20.3 (18.0–22.8)	8.6 (7.0–10.5)	9.3 (7.6–11.2)	1.0 (0.5–1.8)	0.9 (0.4–1.7)
Cataract	828	836	13.8 (11.5–16.3)	13.2 (10.9–15.6)	5.1 (3.7–6.8)	6.8 (5.2–8.7)	1.0 (0.4–1.9)	0.8 (0.3–1.7)
Asthma	698	714	15.3 (12.7–18.2)	12.9 (10.5–15.6)	4.4 (3.0–6.2)	4.2 (2.9–5.9)	2.3 (1.3–3.7)	2.5 (1.5–4.0)
Respiratory disorders^#^	651	593	20.0 (17.0–23.3)	23.1 (19.8–26.7)	13.4 (10.8–16.2)	15.0 (12.2–18.1)	0.6 (0.2–1.6)	2.0 (1.0–3.5)
Renal disorders	328	319	26.2 (21.5–31.3)	23.5 (19.0–28.6)	15.2 (11.5–19.6)	15.7 (11.9–20.1)	0.6 (0.1–2.2)	1.3 (0.3–3.2)
**Number of selected medical conditions present at enrollment**								
1	3,309	3,333	6.9 (6.0–7.8)	7.7 (6.8–8.7)	3.2 (2.6–3.9)	3.4 (2.8–4.0)	1.4 (1.0–1.9)	1.2 (0.9–1.7)
2	3,267	3,238	9.7 (8.7–10.8)	9.7 (8.7–10.8)	3.9 (3.3–4.6)	4.5 (3.8–5.3)	1.3 (1.0–1.8)	1.6 (1.2–2.1)
3	2,449	2,567	12.0 (10.7–13.3)	13.0 (11.7–14.3)	5.6 (4.7–6.5)	5.8 (5.0–6.8)	1.2 (0.8–1.7)	1.2 (0.8–1.7)
At least 3	5,487	5,555	14.7 (13.8–15.7)	14.8 (13.9–15.8)	6.4 (5.8–7.1)	6.7 (6.0–7.3)	1.2 (0.9–1.5)	1.5 (1.2–1.8)
At least 4	3,038	2,988	16.9 (15.6–18.3)	16.4 (15.1–17.7)	7.1 (6.3–8.1)	7.4 (6.5–8.4)	1.2 (0.8–1.6)	1.7 (1.2–2.2)
At least 5	1,490	1,430	19.8 (17.8–21.9)	21.0 (19.0–23.3)	8.6 (7.2–10.1)	9.5 (8.0–11.2)	1.1 (0.7–1.8)	1.6 (1.0–2.4)
At least 6	600	591	21.5 (18.3–25.0)	25.2 (21.8–28.9)	10.2 (7.9–12.9)	11.3 (8.9–14.2)	1.2 (0.5–2.4)	1.4 (0.6–2.6)

RZV = participants receiving the adjuvanted herpes zoster subunit vaccine; Placebo = participants receiving placebo; ZOE-50/70 = RZV efficacy studies in adults ≥50 YOA (NCT01165177) and ≥70 YOA (NCT01165229), respectively; % = percentage of participants in the category; CI = confidence interval; pIMDs = potential immune-mediated diseases; SAEs = serious adverse events; TVC = total vaccinated cohort.

^#^Other than asthma.

## Discussion

Older adults enrolled in the ZOE-50/70 studies reported underlying medical conditions at a frequency expected in these age groups.^11^ The observed efficacy across the 15 medical conditions, including the ones associated with an increased HZ risk, was consistent with the efficacy in the overall pooled ZOE-50/70 population ≥70 YOA.^8^ This is in line with the fact that >80% of the overall ZOE-50 and ZOE-70 study population reported at least 1 of the specified conditions. Other studies have shown that RZV also confers strong protection against HZ in immunocompromised populations who are at highest HZ risk, such as hematopoietic stem cell transplant recipients and patients with hematological malignancies.^12,13^

The high proportion of participants reporting at least 1 of the selected medical conditions can be explained by the relatively permissive inclusion and exclusion criteria for the ZOE-50/70 studies and the age of study participants. Previous studies have shown that frailty correlates with the number, rather than the nature of accumulated biopsychosocial deficits, which are mostly medical conditions.^14,15^ Our analysis was consistent with this finding in that the frequency of SAEs and deaths increased with the number of conditions reported at enrollment. Although the point estimates for efficacy were <90% for 5 of the 15 selected medical conditions, the observed trend suggest that a high VE was maintained in participants having up to 6 or more of those conditions. This contrasts with the observed decline in influenza vaccination effectiveness as the level of frailty increases in elderly people.^16^ This further underscores the ability of RZV to induce robust protection against HZ in older adults with multiple medical conditions.

Overall, frequencies of SAEs, deaths, and pIMDs were balanced between RZV and placebo recipients within each of the analyzed sub-groups. No vaccine-related safety concerns were identified in participants with any type or number of the selected medical conditions.

This post-hoc analysis has some limitations that should be taken into account when interpreting its results. The ZOE-50/70 studies were not statistically powered to evaluate RZV efficacy and safety by the number and type of participants’ medical conditions. In addition, the number of participants in some sub-groups was limited. This includes medical conditions reported by less than 500 participants in the ZOE-70 study at enrollment but associated with an increased risk of HZ (asthma, depression, respiratory disorders, and renal disorders) and participants with ≥6 of the selected conditions. The present analysis was not adjusted for multiplicity, and being exploratory, its significance level was not controlled. The efficacy and safety analyses by medical condition category did not detect any additional underlying medical condition at enrollment that might have an additive or synergistic effect on the risk of HZ. As the study groups were comparable as a result of randomization, this should have a limited effect on the analyses by type or number of conditions. In addition, study participants were not fully representative of the overall older adult population. Individuals with a life expectancy of less than 4 years at the time of study entry were to be excluded. Persons with diseases requiring treatment with immune-modifying drugs, as well as persons with diseases that are immunosuppressive by nature, were also excluded, limiting the conditions for which we could provide a VE estimate. Therefore, owing to the inclusion/exclusion criteria, persons with some of the conditions associated with the highest HZ risk were not enrolled in the study. Some of these conditions were studied as part of the parallel clinical program with RZV in immune compromised adults (e.g. hematopoietic stem cell transplant recipients and patients with hematological malignancies). No standard definitions were used in the diagnosis; therefore, each selected medical condition could vary with respect to severity, stage, treatment, progression, or type (e.g., diabetes mellitus type). The database did not capture this level of detail. Medical conditions with onset after enrollment were not considered for this analysis. Older adults with severe frailty (e.g., bedridden elderly persons) were also unlikely to participate. Despite their broad geographic diversity, study participants were mostly Caucasian. Nonetheless, the most common diseases in the targeted age group were well represented.

In summary, this study showed that >80% of participants had at least 1 of the 15 selected medical conditions present at enrollment. As indicated by the similarity of the point estimates, this post-hoc analysis suggests that RZV efficacy remains high in all selected medical conditions, as well as with increasing number of medical conditions. Point estimates for efficacy ranged between 84.5–97.0% according to the type of the selected medical conditions (with overlapping 95% confidence intervals) and were >90% even among participants reporting at least 6 of these at enrollment. No safety concern was identified in adults ≥50 YOA presenting these medical conditions.

A plain language summary contextualizing the results and potential clinical research relevance and impact is displayed in the Focus on Patient Section (Supplementary Figure 1).

## ZOE-50/70 study group

Anitta Ahonen, Eugene Athan, Johan Berglund, Won Suk Choi, Ferdinandus J. de Looze, Maria Giuseppina Desole, Meral Esen, Brecht Geeraerts, Wayne Ghesquiere, Lars Rombo, Antonio Volpi.

## Trademark statement

Shingrix is a trademark owned by or licensed to the GSK group of companies.
