# Supplementary material for: Medical conditions at enrollment do not impact efficacy and safety of the adjuvanted recombinant zoster vaccine: a pooled post-hoc analysis of two parallel randomized trials
Source: Hum Vaccin Immunother. 2019 Jun 28;15(12):2865–72. doi: 10.1080/21645515.2019.1627818 (PMC6930113; doi:10.1080/21645515.2019.1627818)

**Supplementary materials**

**Members of the ZOE-50/70 study group (by country, in alphabetical order)***

Australia: Eugene Athan, Ferdinandus J. de Looze

Canada: Wayne Ghesquiere

Finland: Anitta Ahonen

Germany: Meral Esen

Italy: Maria Giuseppina Desole, Antonio Volpi

Korea: Won Suk Choi

Sweden: Johan Berglund, Lars Rombo

GSK: Brecht Geeraerts

*Only investigators who agreed to participate in the publication are listed here; therefore, not all countries are represented.

**Authors’ and study group contribution (by alphabetical order)**

The list of contributors who conceived and designed separately the ZOE-50 and ZOE-70 studies, as well as those who collected and interpreted the ZOE-50 and ZOE-70 studies data can be found in the contributor sections here [7, 8].

*Generated the post-hoc analyses data:* Alemnew F. Dagnew, Brecht Geeraerts, Caroline Hervé, George Kalema, Tomas Mrkvan, Lidia Oostvogels, Peter Van den Steen, and Toufik Zahaf.

*Interpreted the post-hoc analyses data:* Roman Chlibek, Anthony L. Cunningham, Alemnew F. Dagnew, Javier Diez-Domingo, Iris S. Gorfinkel, Thomas C. Heineman, Caroline Hervé, Shinn-Jang Hwang, Hideyuki Ikematsu, Robert W. Johnson, George Kalema, Himal Lal, Myron J. Levin, Janet E. McElhaney, Shelly A. McNeil, Tomas Mrkvan, Lidia Oostvogels, Karlis Pauksens, Jan Smetana, Peter Van den Steen, Daisuke Watanabe, Lily Yin Weckx, and Toufik Zahaf.

*Wrote the manuscript (Core Writing Team):* Anthony L. Cunningham, Alemnew F. Dagnew, Thomas C. Heineman, Robert W. Johnson, Myron J. Levin, Janet E. McElhaney, Lidia Oostvogels, Peter Van den Steen, and Toufik Zahaf. All authors reviewed and approved the final submitted version of the paper. All group contributors had the opportunity to review a draft of the paper.

**Supplementary table 1.** Grouping of MedDRA PTs

| **Medical condition category** | **MedDRA PT** | **MedDRA SOC** |
| --- | --- | --- |
| Hypertension | Hypertension | Vascular disorders |
|  | Essential hypertension |  |
| Osteoarthritis and/or vertebral disorders | Osteoarthritis | Musculoskeletal and connective tissue disorders |
|  | Arthralgia |  |
|  | Arthritis |  |
|  | Back pain |  |
|  | Spinal osteoarthritis |  |
|  | Intervertebral disc protrusion |  |
|  | Intervertebral disc degeneration |  |
|  | Spinal column stenosis |  |
|  | Lumbar spinal stenosis |  |
|  | Spinal pain |  |
|  | Lumbar radiculopathy | Nervous system disorders |
| Dyslipidemia | Hypercholesterolemia | Metabolism and nutrition disorders |
|  | Hyperlipidemia |  |
|  | Dyslipidemia |  |
| Diabetes | Type 2 diabetes mellitus | Metabolism and nutrition disorders |
|  | Diabetes mellitus |  |
|  | Type 1 diabetes mellitus |  |
|  | Insulin-requiring type 2 diabetes mellitus |  |
| Osteoporosis/Osteopenia | Osteoporosis | Musculoskeletal and connective tissue disorders |
|  | Osteopenia |  |
| Gastroesophageal reflux disease | Gastroesophageal reflux disease | Gastrointestinal disorders |
| Sleep disorder | Insomnia | Psychiatric disorders |
|  | Sleep disorder |  |
| Prostatic diseases | Benign neoplasm of prostate | Neoplasms benign, malignant and unspecified (incl cysts and polyps) |
|  | Neoplasm prostate |  |
|  | Prostatic adenoma |  |
|  | Prostate cancer |  |
|  | Prostate cancer stage II |  |
|  | Benign prostatic hyperplasia | Reproductive system and breast disorders |
|  | Prostatic disorder |  |
|  | Prostatic dysplasia |  |
|  | Prostatism |  |
|  | Prostatomegaly |  |
| Hypothyroidism | Hypothyroidism | Endocrine disorders |
| Depression | Depression | Psychiatric disorders |
| Coronary heart disease | Coronary artery disease | Cardiac disorders |
|  | Myocardial ischemia |  |
| Cataract | Cataract | Eye disorders |
| Asthma | Asthma | Respiratory, thoracic and mediastinal disorders |
| Respiratory disorders^#^ | Chronic obstructive pulmonary disease | Respiratory, thoracic and mediastinal disorders |
|  | Bronchitis chronic |  |
|  | Obstructive airways disorder |  |
| Renal disorders | Chronic kidney disease | Renal and urinary disorders |
|  | Renal failure |  |
|  | Renal impairment |  |

MedDRA = Medical Dictionary for Regulatory Authorities; PT = preferred term; SOC = system organ class

^#^Other than asthma.

**Supplementary figure 1.** Focus on the patient


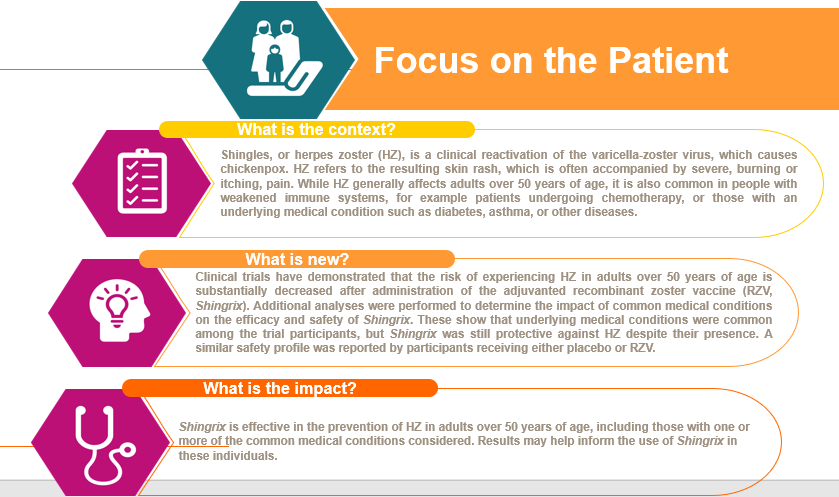

Supplement: Supplemental Material [file khvi-15-12-1627818-s001.docx]
